# Psycho-Socioeconomic Factors Affecting Complementary and Alternative Medicine Use among Selected Rural Communities in Malaysia: A Cross-Sectional Study

**DOI:** 10.1371/journal.pone.0112124

**Published:** 2014-11-06

**Authors:** Kurubaran Ganasegeran, Anantha Kumar Rajendran, Sami Abdo Radman Al-Dubai

**Affiliations:** 1 Medical Department, Tengku Ampuan Rahimah Hospital (HTAR), Jalan Langat, Klang, Selangor, Malaysia; 2 International Medical School, Management and Science University (MSU), Off Persiaran Olahraga, Section 13, Shah Alam, Selangor, Malaysia; 3 Department of Community Medicine, International Medical University (IMU), No. 126, Jalan Jalil Perkasa 19, Bukit Jalil, Kuala Lumpur, Malaysia; The National Institute for Health Innovation, New Zealand

## Abstract

**Introduction:**

The use of complementary and alternative medicine (CAM) as a source of cure has gained much spectrum worldwide, despite skeptics and advocates of evidence-based practice conceptualized such therapies as human nostrum.

**Objective:**

This study aimed to explore the factors affecting CAM use among rural communities in Malaysia.

**Methods:**

A cross-sectional study was carried out on 288 occupants across four rural villages within the District of Selama, Perak, Malaysia. A survey that consisted of socio-economic characteristics, history of CAM use and the validated Holistic Complementary and Alternative Medicine Questionnaire (HCAMQ) were used.

**Results:**

The prevalence of self-reported CAM use over the past one year was 53.1%. Multiple logistic regression analyses yielded three significant predictors of CAM use: monthly household income of less than MYR 2500, higher education level, and positive attitude towards CAM.

**Conclusion:**

Psycho-socioeconomic factors were significantly associated with CAM use among rural communities in Malaysia.

## Introduction

*“Natural forces within us are the true healers of disease.”*

*Hippocrates, 460 BC – ca. 370BC*

While modern revolutionary medicine failed to cure and prevent populations being plagued with debilitating non-communicable diseases like cancer, diabetes and hypertension, efforts prompted by complementary and alternative medicine (CAM) healers through holistic philosophical orientation that focuses the triadic natural forces of mind, body and soul as sources of cure has gained much spectrum worldwide [1]–[3]. Skeptics and advocates of evidence-based practice conceptualized such therapies as human nostrum [4]. The prevalence of CAM use in some developed nations varied between 30 and 90%, with large scale surveys suggesting an increasing popularity of CAM use in North America, Australia and Europe in recent decades [5]. The prevalence of CAM use in some developing countries was almost 70%, with the bulk of populations originating from Asia [2], [6]. In Malaysia, the prevalence of CAM use was approximately 56% yearly [7].

The exponential growth and demand of CAM in Malaysia prompted the establishment of the National Traditional and Complementary Medicine Policy in 2001, allowing a provision to establish ten integrated public hospitals of CAM use within the conventional Malaysian healthcare system under the Ministry of Health Malaysia in 2006 [7]. A CAM integrated hospital facilitates selected CAM practices such as traditional massage and acupuncture for chronic and post stroke management, herbal therapy as an adjuvant treatment for cancer and postnatal care with an aim to ensure the safety and quality of CAM for its clients in addition to the conventional Western medicine practiced in Malaysia [8]. Local and foreign CAM practitioners offering their services in Malaysia's integrated hospitals are required to register with the Traditional and Complementary Medicine (TCM) division under Ministry of Health Malaysia. Practitioners are bound to comply with the proposed guidelines in order to offer evidence based practices. The Standard Operating Procedure (SOP) to conduct CAM services in the hospital set-up requires patients to be seen by the allopathic practitioners before seeking traditional therapies. At present CAM treatment are provided only as outpatient services [8].

CAM's heterogeneity consist of therapeutic practices and products (including acupuncture, chiropractic, naturopathy, traditional herbal medicine and yoga) that are not associated with conventional medical profession [5], but reflecting a different understanding of biological processes [2]. Numerous studies postulated psycho-socioeconomic elements like high education level, gender, age, income level, occupation, poor health status, holistic health beliefs, attitude towards CAM, and associated geographical, culture or political variations across regions as predictive factors for CAM use [2], [5], [9].

Available studies from different countries explored the use of CAM in metropolitan cities, general populations and patients with chronic diseases [5], [6], [10], [11] with limited focus in rural dwellings [10]. Sparse exploration on CAM use in rural areas showed mixed variations, with poor access to conventional health services posit significant barriers for improvement of individuals' health status [5], [10]. In addition, localized community beliefs in folk remedies posed substantial culture-centric sensitivity of pre-existing tenets amongst rural occupants [5]. While the omission of studies in rural communities was perplexing [10], this study aimed to determine the factors affecting CAM use among selected rural communities, from the Malaysian perspective.

## Methods

### Ethics statement

This research protocol was approved by the Ethics Committee of Management and Science University (MSU) and the local District Council of Selama, Perak, Malaysia. A written consent was obtained from those who agreed to participate.

### Study setting and population

This cross-sectional study recruited 288 occupants in the rural region of the Malaysian Northern Development Corridor. The Malaysian Northern Development Corridor was established under the Northern Corridor Implementation Authority Act 2008 (Act 687) to provide direction, policies and strategies in relation to socio-economic development, encompassing 21 districts in northern Peninsular Malaysia within the states of Perak, Kedah, Perlis and Penang [12]. Four out of eight villages were selected randomly from the District of Selama, Perak. We obtained the number of houses from each village to calculate the number of houses for inclusion in the study by proportional stratified sampling. In the third stage we obtained the list of houses in each village and selected the required number of houses by using proportional random sampling. The total sample size was calculated to be 334 based on the 32% expected prevalence rate of CAM use and 0.05 level of accuracy [5]. The response rate was 86% (288/334).

Eligible respondents were Malaysian citizens of at least 18 years of age. All study participants were volunteers of acceptable literacy who could establish verbal and written communications. Trained interviewers approached the occupants across the four villages on a house to house visit. One participant from each household was approached. Objectives and benefits of the study were explained in verbal and written form was attached to the questionnaires in a sealed envelope. Respondent confidentiality and anonymity were assured. The completed self-administered questionnaires were re-collected by the trained interviewers in a sealed envelope from the respondents. Those who refused to participate (27) and those who were approached but aged less than eighteen years old (19) were excluded from the study. None of the participants were illiterate.

### Study instruments

A self-administered questionnaire consisting of three parts was used in this study. The first part included socio-economic characteristics of the respondents (gender, age, education level, occupation and income level). CAM was defined as ‘a group of therapeutic and diagnostic disciplines that exist largely outside the institutions where conventional health care is taught and provided’ [13]. To explore respondents psychological attributes towards CAM, we used the validated Holistic Complementary and Alternative Medicine Questionnaire (HCAMQ), originally developed by Hyland et al., (2003) [14]. HCAMQ is comprised of eleven items, five of which measures holistic health beliefs and another six measures attitude towards CAM. These items are scored on a six-point Likert scale ranging from one (strongly agree) to six (strongly disagree). After reversing the codes of the negatively worded items (items 2, 4, 6 and 9), item scores were summed to provide the total sub-scaled scores of holistic health belief that ranged 1–30 and the total sub-scaled scores of attitude towards CAM that ranged 1–36. The total score of both sub-scales ranged 1–66 [14]. Median cut-offs were obtained for each sub-scale score. A median cut-off point of >10 suggests positive holistic health beliefs, while a median cut-off point of >27 suggests positive attitudes towards CAM. The questionnaire was first designed in English and administered in Malay. The questionnaire was a forward-backward translation to Malay language. The reliability and factor structure analysis showed good psychometric properties. We made a pilot study on 10 occupants from a rural area to ensure that the questionnaire was easily understood and clear. The data collectors were trained before they went to the field. Example of items on attitude towards CAM include “complementary medicine should be subject to more scientific testing before it can be accepted by conventional doctors” and “complementary medicine can be dangerous in that it may prevent people getting proper treatment”, while example of items related to holistic health beliefs include “positive beliefs and spirituality can help you fight off an illness” and “when people are stressed, it is important that they are careful about other aspects of their lifestyle (eg. healthy eating) as their body already has enough to cope with” [14].

### Statistical analysis

Analysis was performed using Statistical Package of Social Sciences (SPSS version 16.0). To check for internal consistency of the Holistic Complementary and Alternative Medicine Questionnaire (HCAMQ) among Malaysian population, an exploratory factor analysis was performed using principal component method with varimax rotation and Cronbach's alpha was used. Descriptive statistics were obtained for all variables in the study. We used cross tabulation analysis to obtain the proportions. Simple logistic regression analysis was used to obtain the odds ratio (OR) and to assess the association between CAM use and categorical variables in this study. Multiple logistic regression analysis using “Enter” technique was performed to obtain the final model and the adjusted odds ratio. All independent variables with significant associations of CAM use in bivariate analysis were included in the multivariate analysis (variables entered were gender, age, education level, occupation, income level, holistic health belief and attitude towards CAM). Multi-collinearity between independent variables was checked by the values of standard errors (SE) not exceeding 5 [15]. The accepted level of significance in this study was set below 0.05 (p<0.05).

## Results

### Sample characteristics and CAM use

The sample consisted of 59% men and 41% women. The average age was 37.5 years (SD = 16.4) and the age ranged from 18 to 80 years. Of the total respondents, 60.1% had tertiary education and 59.7% were employed. The majority had a monthly household income of less than MYR 2500 (84.0%). Most respondents perceived positive holistic health beliefs (71.5%) and the majority exhibited positive attitudes towards CAM use (54.2%) (Table 1).

**Table 1:** Socio-economic and psychological characteristics of respondents (n = 288).

Characteristics		N	%
**Socio-economic Factors**			
	**Gender**		
	Male	170	59.0
	Female	118	41.0
	**Age (years)**		
	<30	133	46.2
	≥30	155	53.8
	**Education level**		
	High school or less	115	39.9
	Tertiary education	173	60.1
	**Occupation**		
	Employed	172	59.7
	Unemployed*	116	40.3
	**Income level (MYR)**		
	<2500	242	84.0
	≥2500	46	16.0
**Psychological Factors**	**Holistic health belief**		
	Negative	82	28.5
	Positive	206	71.5
	**Attitude towards CAM**		
	Negative	132	45.8
	Positive	156	54.2

*Unemployed is defined as those without a paid job.

Cronbach's alpha coefficient for holistic health belief subscale was 0.83, showing a high internal consistency, while Cronbach's alpha coefficient for attitude towards CAM subscale was 0.74, showing an acceptable internal consistency. The exploratory factor analysis yielded two factors with initial Eigenvalues greater than 1. The six items that measure the attitude towards CAM loaded on the first factor and the five items that measure the holistic health beliefs loaded on the second factor (Table 2). Five therapies were used by more than 10 per cent of the sample (Table 3).

**Table 2:** Factor structure of the Holistic Complementary and Alternative Medicine Questionnaire (HCAMQ).

Item	Statement	Factor 1	Factor 2
1	Positive beliefs and spirituality can help you fight off an illness.	0.458	0.326
2	Complementary medicine should be subject to more scientific testing before it can be accepted by conventional doctors.	0.176	0.630
3	When people are stressed, it is important that they are careful about other aspects of their lifestyle (eg. healthy eating) as their body already has enough to cope it.	0.822	0.117
4	Complementary medicine can be dangerous in that it may prevent people getting proper treatment.	0.067	0.709
5	The symptoms of an illness can be made worse by depression.	0.650	0.292
6	Complementary medicine should only be used as a last resort when conventional medicine has nothing to offer.	0.235	0.785
7	If a person experiences a series of stressful life events they are likely to become ill.	0.795	0.188
8	It is worthwhile trying complementary medicine before going to the doctor.	0.105	0.699
9	Complementary medicine should only be used for minor ailments and not for the treatment of more serious illness.	0.117	0.728
10	It is important to find a balance between work and relaxation in order to stay healthy.	0.717	0.222
11	Complementary medicine builds up the body's own defenses, so leading to a permanent cure.	0.149	0.654

**Table 3:** Use of CAM among respondents (n = 288).

CAM Modalities	n	%
Traditional Malay Medicine (*Malay herbs, Urut Melayu, Bekam*)	98	34.0
Traditional Chinese Medicine (*Chinese herbs, Acupuncture, T'ai Chi, Qi Gong*)	32	11.1
Traditional Indian Medicine (*Ayurveda, Siddha, Unani, Yoga/Meditation*)	46	16.0
Manipulative Based Practice (*Chiropractor, Osteopathy, Massage, Spa therapy, Reflexology, Aromatherapy*)	45	15.6
Energy Medicine (*Reiki/Therapeutic touch, Crystal healing, Aura metaphysics*)	33	11.5
Mind, Body & Soul Therapy (*Hypnotherapy, Psychotherapy*)	28	9.7
Biological Based Medicine (*Nutritional therapy, Naturopathy*)	21	7.3
Homeopathy	24	8.3

### Association between CAM use and psycho-socioeconomic factors among respondents

The use of CAM was significantly higher in males (52.4%) compared to females (OR = 1.7, 95% CI 1.1–2.8, p = 0.025). Those aged more than 30 years old (66.5%) had a significantly higher use of CAM compared to younger ones (37.6%) (OR = 3.3, 95% CI 2.0–5.3, p<0.001). Those attained tertiary education (54.9%) had a significantly higher use of CAM compared to high school graduates or less (34.8%) (OR = 2.5, 95% CI 1.4–3.3, p = 0.001). Unemployed occupants (55.2%) had a significantly higher use of CAM in comparison to those being employed (OR = 1.7, 95% CI 1.1–2.5, p = 0.020). Occupants with a monthly household income of less than MYR 2500 (52.5%) had a significantly higher use of CAM compared to those with higher income level (17.4%) (OR = 5.2, 95% CI 2.4–11.7, p<0.001). The use of CAM was significantly higher among occupants with a positive holistic health beliefs (53.4%) compared to those with negative beliefs (30.5%) (OR = 2.5, 95% CI 1.4–5.0, p<0.001). Occupants with positive attitudes towards CAM (62.2%) had a significantly higher use of CAM compared to those with negative attitudes (28.8%) (OR = 5.0, 95% CI 2.5–10.0, p<0.001) (Table 4).

**Table 4:** Association between CAM use and psycho-socioeconomic factors in the simple logistic regression analysis (n = 288).

Characteristics		Yes n (%)	No n (%)	OR	95% CI	P-value
**Socio-economic Factors**						
	**Gender**					
	Male	89 (52.4)	81 (47.6)	1.7	1.1–2.8	0.025
	Female	46 (39.0)	72 (61.0)	1		
	**Age (years)**					
	<30	50 (37.6)	83 (62.4)	1		
	≥30	103 (66.5)	52 (33.5)	3.3	2.0–5.3	<0.001
	**Education level**					
	High school or less	40 (34.8)	75 (65.2)	1		
	Tertiary education	95 (54.9)	78 (45.1)	2.5	1.4–3.3	0.001
	**Occupation**					
	Employed	71 (41.3)	101 (58.7)	1		
	Unemployed	64 (55.2)	52 (44.8)	1.7	1.1–2.5	0.020
	**Income level (MYR)**					
	<2500	127 (52.5)	115 (47.5)	5.2	2.4–11.7	<0.001
	≥2500	8 (17.4)	38 (82.6)	1		
**Psychological Factors**						
	**Holistic health belief**					
	Negative	25 (30.5)	57 (69.5)	1		
	Positive	110 (53.4)	96 (46.6)	2.5	1.4–5.0	<0.001
	**Attitude towards CAM**					
	Negative	38 (28.8)	94 (71.2)	1		
	Positive	97 (62.2)	59 (37.8)	5.0	2.5–10.0	<0.001

### Factors affecting CAM use among respondents by multiple logistic regression analyses

Multiple logistic regression analyses yielded three significant predictors of CAM use. Monthly household income of less than MYR 2500 was the most significant predictor of CAM use in the model (OR = 9.5, 95% CI 4.0–22.8, P<0.001), followed by positive attitude towards CAM (OR = 3.7, 95% CI 2.1–6.4, P<0.001) and higher education level (OR = 2.4, 95% CI 1.4–4.3, P = 0.003). The total model was significant (P<0.001) and accounted for 25% of the variance (Table 5).

**Table 5:** Multiple logistic regression analysis (Backward Wald); factors affecting CAM use among respondents (n = 288).

Predictors	B	SE	Wald	Exp (B)*	95% CI	P value
**Education level**						
High school or less	Ref	Ref	Ref	Ref	Ref	Ref
Tertiary education	0.9	0.3	9.0	2.4	1.4–4.3	0.003
**Income level (MYR)**						
<2500	2.3	0.4	25.7	9.5	4.0–22.8	<0.001
≥2500	Ref	Ref	Ref	Ref	Ref	Ref
**Attitude towards CAM**						
Negative	Ref	Ref	Ref	Ref	Ref	Ref
Positive	1.3	0.3	21.0	3.7	2.1–6.4	<0.001

*Exp (B) gives the Odds Ratio.

## Discussion

This study is an exploratory study that aimed to show the magnitude of CAM use in the population. The finding of this study may encourage authorities to explore this issue and to investigate about the types of CAM used and its meeting with the regulatory issues in the country. Of the 288 respondents surveyed, 53.1% used CAM over the past one year. The prevalence of CAM use in the present study was similar than that found among HIV carriers in Thailand [6], but relatively higher than that found among rural communities with diabetes in USA [16], [17]. The final regression model yielded three factors of education level, income level and attitude towards CAM as significant predictors of CAM use among rural occupants. Income level was the most important factor to influence CAM use among rural occupants in Malaysia.

The association between CAM use and socio-economic factors reserved complexity by previous studies for further exploration [18]. CAM use was significantly higher among males than females in this study, inconsistent with previous studies that reported otherwise [18], [19]. This study found a significantly higher rate of CAM use among those aged more than 30 years old. Similarly, previous studies demonstrated higher use of CAM among older adults due to greater vulnerability of chronic diseases and poor health [20], [21]. McLaughlin et al., (2012) [22] found that older adults expressing dissatisfaction in conventional medicine due to failure of symptoms control and influence of traditional rural values such as self-reliance, individualism and reluctance to seek medical care at early stage of the disease had higher use of CAM.

This study found significant associations between occupation, income level and CAM use. Unemployed occupants and those with lower income had significantly higher use of CAM. Similar findings were found in previous studies [3], [17], [18]. Cost of seeking treatment was an incentive for the use of CAM [18], as most CAM user preferred relatively inexpensive methods such as traditional herbal remedies in rural and remote areas of Malaysia [7].

Most populations using CAM have the highest educational level [23]. In Asia, education and cultural influence are interconnected, with highly educated populations being more ‘culture creative persons’ in exploring CAM [23]. This study found a significantly higher rate of CAM use among respondents with tertiary education compared to high school graduates. Similar findings were reported in other studies [23], [24].

Holistic health belief reflects an emphasis on treating the whole person (rather than symptomatic approach) by using CAM [25]. This study found that the use of CAM was significantly higher among respondents with positive holistic health beliefs. Similar findings were reported in previous studies [23], [25], [26]. Astin (1998) [23] postulated that cultural groups prefer unconventionality and committed to elements like feminism, environmentalism, spirituality, personal growth and love towards the foreign and exotic. A multicultural Malaysia with diversified religions and spiritual beliefs would support the current findings, as strong spiritual ethos necessitates wider belief systems for CAM use [25].

Attitude of one's health self-care contributes to CAM use. A positive attitude towards CAM catalyzes an internal health locus of control as fundamentals of good health are dynamic equilibrium between holistic health beliefs and personal behavior for seeking CAM treatments [27]. This study found that the use of CAM was significantly higher among respondents with positive attitudes toward CAM. McFadden et al., (2010) [27] reported similar findings.

Ensuring safety, efficacy and quality of CAM products is a core objective of World Health Organization (WHO) towards recognizing the role of CAM in modern health care system. Research activities in Malaysian CAM policy are focused to evidence based medicines. Clinical expertise, research evidence and patient's values and preferences are identified as key components of evidence based practice [8]. To date, several publications such as ‘Guidelines on Malay massage and postnatal care, acupuncture, reflexology’ and ‘Herbal therapies as an adjunct treatment for cancer’ have been developed and readily available for both traditional and conventional practitioners who intend to offer CAM practices [8].

Concerns arise on issues related to herbal safety, adulteration, therapeutic approaches, training requirements and professional ethics of CAM practices needs to be addressed urgently [8], [28]. Focus on evidence based practice would assist providers to evaluate clinical effectiveness of CAM treatment while eliminating unsafe practices [8]. The integration of CAM within the conventional health care system requires significant research efforts from the clinical and behavioral aspects [8], [28].

## Limitations

The relatively small sample size from a single geographical area and the absence of multi-ethnic group approach limits the generalizability of the study findings. The relatively large odds ratios (ORs) and wide 95% CI obtained for some outcomes may be due to ‘sparse data’, thus the interpretation of these findings should be interpreted cautiously. Another limitation of this study was that the non-response rate could not be compensated due to budget limitation. Unavailable data about the non-respondent such as socio-demographics and literacy is an additional limitation in this study. The cross-sectional design of this study limits our ability to make causal inferences. Further research is needed to address these limitations.

## Conclusion

Psycho-socioeconomic factors were significantly associated with CAM use among rural communities in Malaysia.

## Recommendations

The need to further investigate other user attributes like local influences of cultural beliefs or informal community networks is essential given that growing evidence of CAM use among Malaysian population. Provision under the Malaysian Medical Act that regulates CAM practice should generate further flexibility to allow increased establishments of CAM integrated hospitals, allowing standardized quality healthcare and prevention of quack practices. The prospect of integrating CAM into conventional medicine in Malaysia is high, as it might turn out to be cost effective with optimal use of time and resources. Efforts by stakeholders in the healthcare sector to generate a flexible and more acceptable health delivery system for integration of CAM into conventional practices would boost economic, social and health well-being.
